# STAG2 loss amplifies EWS-FLI1-driven microsatellite enhancer activity promoting Ewing sarcoma aggressiveness

**DOI:** 10.1073/pnas.2537425123

**Published:** 2026-04-08

**Authors:** Sanjana Eyunni, Shih-Chun Chu, Mary L. Guan, Michaela Louw, Eleanor Young, Sandra E. Carson, Jianhui Gong, Marcin Cieslik, Arul M. Chinnaiyan, Abhijit Parolia

**Affiliations:** ^a^Department of Pathology, University of Michigan, Ann Arbor, MI 48109; ^b^Michigan Center for Translational Pathology, University of Michigan, Ann Arbor, MI 48109; ^c^Department of Computational Medicine and Bioinformatics, University of Michigan, Ann Arbor, MI 48109; ^d^Rogel Cancer Center, University of Michigan, Ann Arbor, MI 48109; ^e^HHMI, University of Michigan, Ann Arbor, MI 48109; ^f^Department of Urology, University of Michigan, Ann Arbor, MI 48109

**Keywords:** STAG2-cohesin, GGAA repeat enhancers, repeat-length–specific gene signatures, disease prognostication, EWS-FLI1 cistromic reprogramming

## Abstract

Ewing sarcoma is driven by chromosomal translocations that fuse a FET RNA-binding protein to an ETS transcription factor, most commonly generating the EWS-FLI1 fusion oncoprotein. EWS-FLI1 engages GGAA microsatellite repeats to form de novo enhancers that activate oncogenic transcriptional programs essential for tumorigenesis. In addition to this truncal driver, recurrent loss-of-function alterations in the cohesin subunit STAG2 occur in approximately 10 to 15% of Ewing sarcomas and are associated with adverse clinical outcomes. However, how STAG2 loss reshapes EWS-FLI1 chromatin engagement and transcriptional output remains poorly understood. Here, using genetic STAG2 loss-of-function models combined with integrative multiomic profiling, we demonstrate that STAG2–cohesin deficiency reprograms the EWS-FLI1 chromatin landscape by altering its binding at GGAA-microsatellite enhancers. Despite increased EWS-FLI1 protein abundance, STAG2 loss eliminates over 40% of EWS-FLI1 binding sites, predominantly at enhancers containing short (1–4) GGAA repeats, while concurrently increasing binding at multimeric enhancers with ≥5 GGAA-repeat motifs. These reprogrammed sites show changes in both chromatin accessibility and H3K27ac, leading to selective amplification of EWS-FLI1 activity at multimeric microsatellite enhancers. By integrating Hi-C chromatin interaction maps with altered EWS-FLI1 occupancy, we define distinct monomeric and multimeric GGAA enhancer–driven transcriptional gene signatures and demonstrate that STAG2 loss selectively augments the multimeric transcriptional program. Consistently, the long GGAA microsatellite-activated gene signature is enriched in patient tumors with aggressive clinical features and deleterious STAG2 alterations. Together, these findings reveal that STAG2 loss reprograms, rather than globally attenuates, EWS-FLI1 function, amplifying a high-risk oncogenic transcriptional state in Ewing sarcoma.

## Significance

Ewing sarcoma is driven by the EWS-FLI1 fusion transcription factor, which activates cancer-promoting genes through GGAA microsatellite enhancers. Loss of the cohesin subunit STAG2 is a recurrent alteration in aggressive Ewing sarcoma, but its functional consequences have remained unclear. This study shows that STAG2 loss does not simply weaken EWS-FLI1 activity; instead, it remodels the enhancer landscape, shifting EWS-FLI1 binding toward long GGAA microsatellites that drive a high-risk transcriptional state. These findings provide a mechanistic explanation for the poor prognosis associated with STAG2-deficient tumors and identify enhancer architecture as a key determinant of oncogenic transcriptional output in Ewing sarcoma.

Ewing sarcoma is a highly aggressive bone and soft tissue cancer that affects children and young adults, with about 25% of patients presenting with a therapy-refractory metastatic disease and a 5-y survival rate of roughly 30% despite intensive multimodal treatment (1). While Ewing sarcomas have an overall low mutation burden and stable genomes (2–4), they are characterized by chromosomal translocations that fuse members of the FET RNA-binding protein family (e.g., EWSR1, FUS, and TAF15) to an ETS family transcription factor (e.g., FLI1, ERG, and FEV), the most common of which is the EWS-FLI1 fusion oncogene detected in over 90% of the cases (1, 5, 6). Through neomorphic chromatin pioneering activity, EWS-FLI1 demarcates and decompacts de novo enhancers containing GGAA-microsatellite repeat sequences (7, 8), which are functionally activated by fusion-dependent recruitment of the SWI/SNF chromatin remodeling complexes and the histone acetyltransferase p300 (9–11).

In addition to the truncal fusion, recurrent mutations in the STAG2 gene are seen in about 15 to 20% of Ewing sarcoma patients (3, 12), and its loss is strongly associated with metastatic disease and poor overall survival (12, 13). Together with the core cohesin subunits SMC1A, SMC3, and RAD21, STAG2 forms the cohesin ring that mediates sister-chromatid cohesion during cell division (14, 15) and organizes higher-order genomic structure by establishing topologically associating domains (TADs) that are anchored at CTCF binding sites (16–18). Beyond this structural role, cohesin facilitates long-range chromatin looping that connects distal enhancers with target gene promoters, thereby establishing cell-type–specific intra-TAD three-dimensional architecture required for regulation of gene expression (19–21).

Early models proposed STAG2 loss to promote tumorigenesis primarily through defective sister-chromatid cohesion during mitosis and ensuing aneuploidy (22). However, genomic studies focused on urothelial bladder cancer and myeloid malignancies found that most STAG2-deficient tumors remain karyotypically stable (23–25). Similarly, genomic studies focused on Ewing sarcoma uncovered no evidence of increased aneuploidy in STAG2-mutant versus wildtype cases (2, 3), particularly when controlling for coincident TP53 loss-of-function alterations (4). Recent mechanistic studies showed that STAG2 loss in Ewing sarcoma primarily perturbs gene transcription by decreasing enhancer–promoter interactions anchored at a subset of EWS-FLI1 sites, thereby creating an “EWS-FLI1 low” transcriptional state that favors mesenchymal features and metastatic behavior (26–28).

In this study, we extend prior observations and show that the primary pathogenic consequence of STAG2 loss arises from epigenomic enhancer remodeling, rather than chromosomal instability. Through integrative multiomic analyses, we show that depletion of STAG2 redirects EWS-FLI1 binding away from short GGAA elements toward long, multimeric GGAA-microsatellite enhancers. Consequently, STAG2 loss does not merely diminish EWS-FLI1 transcriptional activity; instead, it reprograms it to amplify the EWS-FLI1 transcriptional program regulated through long GGAA-repeat enhancers and promoters, while attenuating genes predominantly driven by short GGAA-repeat control elements. By partitioning EWS-FLI1 transcriptional output based on the GGAA-repeat number within enhancers, we define distinct gene-expression signatures with prognostic significance, reframing the prevailing model of a uniform “EWS-FLI1–low” state into a GGAA repeat-length–dependent reprogramming of EWS-FLI1 oncogenic activity following STAG2 inactivation in Ewing sarcoma.

## Results

### STAG2 Inactivation Shifts EWS-FLI1 Binding Toward Long GGAA Microsatellite Enhancers.

**Fig. 1.**
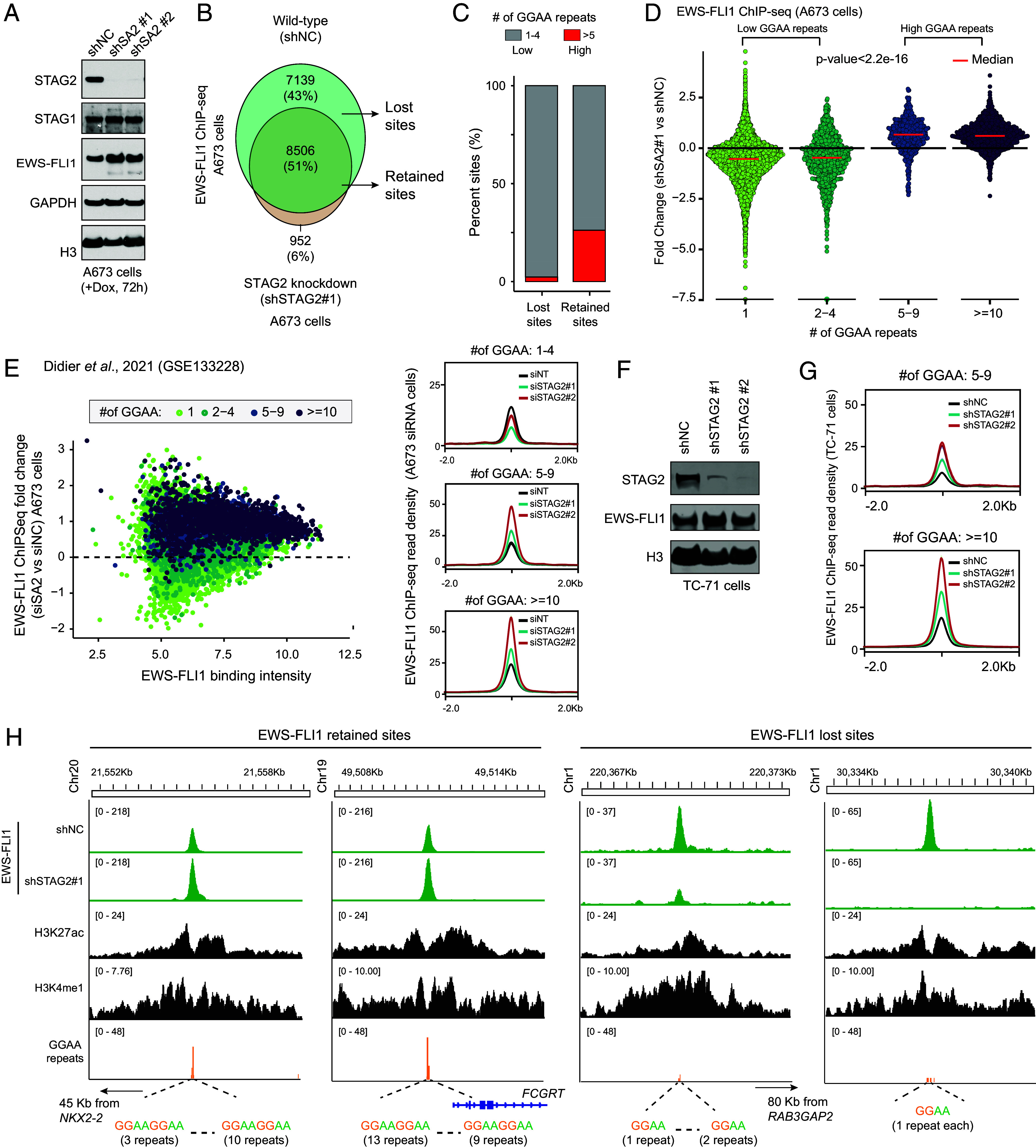
STAG2 loss redirects EWS-FLI1 toward long GGAA-repeat microsatellite enhancers. (*A*) Immunoblot of noted proteins in A673 cells expressing non-targeting control (shNC) or two independent STAG2 shRNAs following 72 h of doxycycline treatment. (*B*) Venn diagram overlap of EWS-FLI1 ChIP-seq peaks between A673 shNC and shSTAG2#1 cells, highlighting sites retained or lost upon STAG2 knockdown. (*C*) Bar plot quantifying the proportion of EWS-FLI1 ChIP-seq peaks with low versus high GGAA repeat number among retained and lost sites in A673 cells. (*D*) Violin plots showing fold change in EWS-FLI1 ChIP-seq signal in A673 control and shSTAG2#1-treated cell lines, stratified by GGAA repeat number. (*E*) Scatter plot comparing EWS-FLI1 ChIP-seq fold change in A673 control or STAG2 siRNA-treated cells as a function of EWS-FLI1 binding intensity, with points colored by GGAA repeat number (28). *Right*: Peak profile plots of EWS-FLI1 ChIP-seq centered on the GGAA microsatellite repeats. (*F*) Immunoblot confirming STAG2 knockdown and EWS-FLI1 expression in TC-71 cells transduced with shNC or two distinct STAG2 shRNAs. (*G*) Peak profile plots of EWS-FLI1 ChIP-seq signal centered on GGAA microsatellites in TC-71 cells transduced with shNC or shSTAG2 shRNAs. (*H*) Genome-browser read density tracks of EWS-FLI1, H3K27ac, and H3K4me1 ChIP-seq signal at representative EWS-FLI1 retained sites (*Left*) and lost (*Right*) sites in A673 cells. The number of tandem GGAA repeats within peak centers is annotated below.

To investigate the impact of STAG2 loss on EWS-FLI1 activity in Ewing sarcoma, we first generated stable doxycycline-inducible shRNA models targeting STAG2 in A673 cells harboring the characteristic EWSR1-FLI1 gene fusion (*SI Appendix,* Fig. S1*A*). Doxycycline treatment efficiently depleted STAG2 protein levels (Fig. 1*A*), which notably had no effect on cell viability or proliferation (*SI Appendix,* Fig. S1*B*). In these models, STAG2 loss resulted in a modest increase in EWS-FLI1 levels, prompting us to examine whether this altered its chromatin occupancy.

Despite an increase in EWS-FLI1 abundance (*SI Appendix,* Fig. S1 *C* and *D*), we uncovered a marked attenuation of the EWS-FLI1 cistrome upon STAG2 inactivation, with over 40% of the sites showing complete loss of binding relative to the control nontargeting shRNA-expressing A673 cells (Fig. 1*B*). Motif analysis of altered sites uncovered a striking shift in the binding preference of EWS-FLI1 depending on the length of GGAA-repeat sequences. In STAG2-deficient cells, we found EWS-FLI1 preferentially lost binding at sites containing short (1–4) GGAA-repeats, whereas binding was retained or gained at sites with longer (≥5) GGAA-repeat sequences (Fig. 1*C*). Stratifying EWS-FLI1 peaks by repeat length further highlighted this pattern: regions harboring 1 to 4× GGAA repeats showed a net loss, while those containing 5 to 9× or ≥10× tandem GGAA repeats exhibited increased binding of EWS-FLI1 fusion protein (Fig. 1*D* and *SI Appendix,* Fig. S1*E*). These findings were reproducible using a second STAG2-targeting shRNA (*SI Appendix,* Fig. S1 *F* and *G*), as well as in independently published EWS-FLI1 ChIP-seq data (28) where STAG2 expression was silenced using siRNA-mediated knockdown (Fig. 1*E*). To confirm these observations in additional Ewing sarcoma models, we engineered inducible shSTAG2 lines of STAG2-wildtype and EWSR1–FLI1 fusion-positive TC-71 cells and analyzed published EWS-FLI1 ChIP-seq data in TC-71 cells (28). Consistent with our findings in A673 cells, depletion of STAG2 levels resulted in a modest increase in EWS-FLI1 expression (Fig. 1*F*), along with a marked increase in its chromatin binding at long GGAA-repeat microsatellite enhancers (Fig. 1*G* and *SI Appendix,* Fig. S1 *H*–*J*).

Finally, to corroborate these effects upon a complete and sustained loss of STAG2-cohesin function, we generated isogenic STAG2-null A673 cells using the CRISPR/Cas9 technique (*SI Appendix,* Fig. S1*K*). Even in these model systems, EWS-FLI1 binding was markedly increased at microsatellite enhancers containing ≥5x GGAA-repeat motifs (*SI Appendix,* Fig. S1 *L*–*N*) compared to STAG2-wildtype control cells. The contrasting effects of STAG2 loss on EWS-FLI1-bound enhancers—reduced binding at short (1 to 4× GGAA) and increased binding at long (≥5× GGAA) microsatellite enhancers—carrying active histone modifications are shown in Fig. 1*H*.

### EWS-FLI1 Redistribution Upon STAG2 Loss Remodels the Chromatin Regulome.

**Fig. 2.**
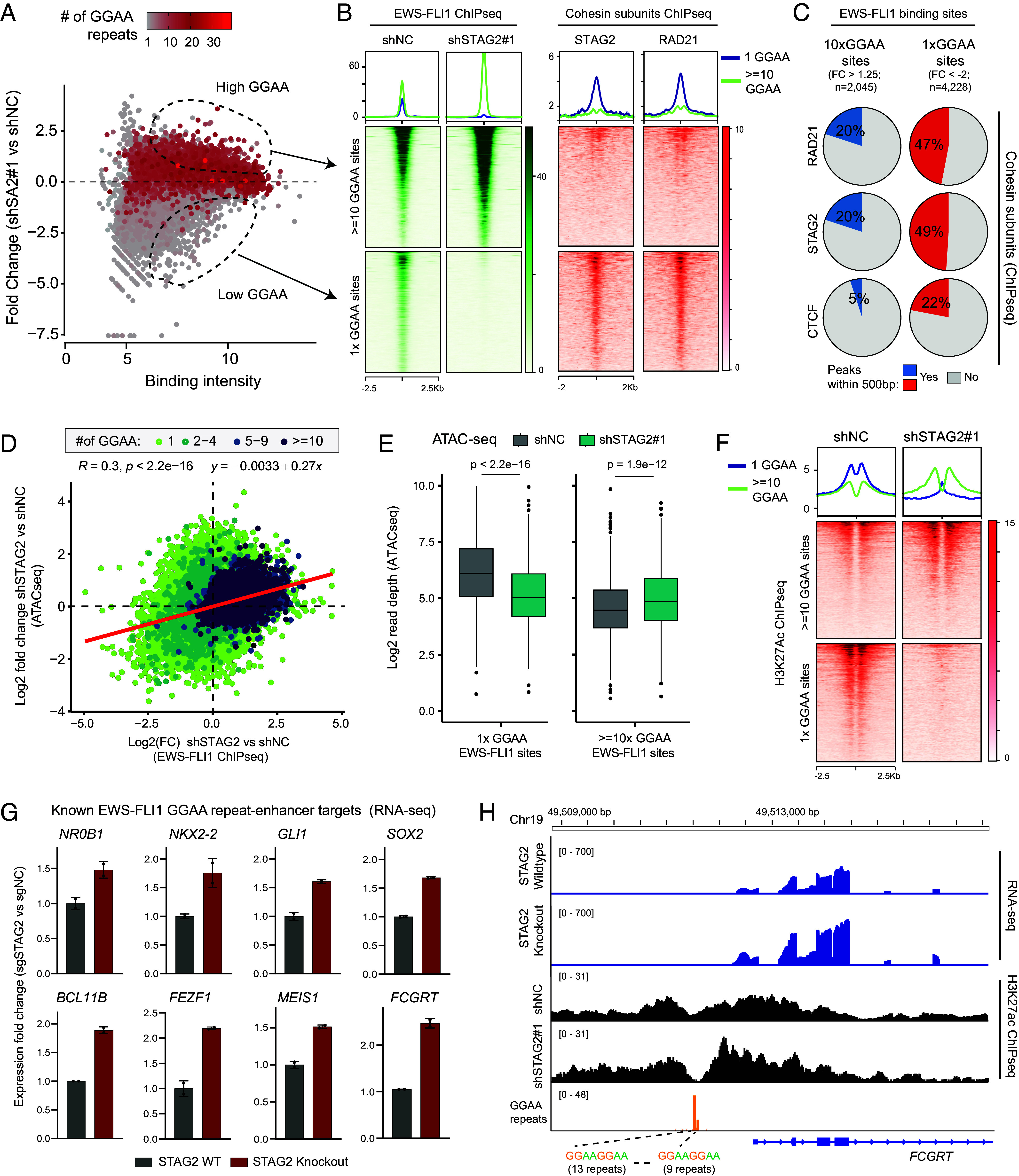
EWS-FLI1 redistribution alters chromatin accessibility and H3K27ac abundance at long GGAA-repeat enhancers. (*A*) Scatter plot comparing EWS-FLI1 ChIP-seq fold change in A673 control or STAG2 shRNA-treated cells as a function of EWS-FLI1 binding intensity, with points colored by GGAA repeat number. (*B*) ChIP-seq read-density heatmaps of EWS-FLI from STAG2 wildtype or deficient A673 cells and cohesin subunits STAG2 and RAD21 from parental A673 cells. (*C*) Pie charts depicting the ChIP-seq binding overlap between EWS-FLI1 and cohesin subunits at 10x and 1x GGAA sites. (*D*) Scatterplot depicting the correlation between fold-change of ChIP-seq and ATAC-seq signal between control or STAG2 shRNA-treated lines. The regression line is shown in red (Pearson correlation test). (*E*) Boxplots of ATAC-seq signal at distinct 1x or 10xGGAA sites in control and STAG2 shRNA-treated lines (n = top 1,000 sites, two-sided *t* test). Box plot center, median; box, quartiles 1 to 3; whiskers, quartiles 1 to 3 ± 1.5× interquartile range; dot, outliers. (*F*) ChIP-seq read-density heatmaps of H3K27ac in A673 control or STAG2 shRNA-treated cells. (*G*) Barplots showing mRNA expression of known FLI1 target genes in A673 control or STAG2 knockout cells (n = 2 biological replicates, RNA-seq; DESeq2). (*H*) Genome-browser read density tracks of RNA-seq and H3K27ac ChIPseq signal at the *FCGRT* gene locus in STAG2-silenced and wildtype control A673 cells.

To characterize the chromatin features associated with altered EWS-FLI1 occupancy following STAG2 loss, we first identified high-confidence enhancer elements that showed a substantial decrease (n = 4,228; fold change<−2) or an increase (n = 2,045; fold change > 1.25) in EWS-FLI1 binding in A673 cells (Fig. 2*A*). Both lost and gained binding sites were predominantly located in distal noncoding intergenic or intronic regions and were marked by H3K27ac and H3K4me1 histone modifications characteristic of active enhancers (*SI Appendix,* Fig. S2 *A* and *B*). Even at these functional elements, over 98% of the lost binding sites contained only 1 to 2 GGAA repeats, whereas all regions showing the strongest gain in EWS-FLI1 binding harbored ≥5xGGAA-repeat sequences (*SI Appendix,* Fig. S2*C*). Remarkably, more than 60% of these gained sites contained ≥15 tandem GGAA sequences centered within the peaks, consistent with EWS-FLI1’s recruitment to microsatellite-type enhancers upon STAG2 loss. In addition, the majority of the gained elements had multiple disconnected ≥5x GGAA blocks, effectively extending the total GGAA-repeat length and density within these regulatory regions (*SI Appendix,* Fig. S2*C*). To assess whether this enhancer reprogramming was conserved across biologically independent disease models, we compared EWS-FLI1 and H3K27ac ChIP-seq data obtained from the Ewing Sarcoma Cell Line Atlas (29) across five STAG2 wild-type (A673, RDES, EW1, EW7, and CHLA10) and four STAG2 mutant cell lines (SKES1, MIC, RH1, and EW22). Consistent with our earlier findings, STAG2 mutant lines showed higher EWS-FLI1 occupancy and stronger functional activation (H3K27ac levels) of multimeric 10xGGAA sites compared to the STAG2 wild-type controls (*SI Appendix,* Fig. S2*D*).

Next, we examined the localization of the cohesin complex near altered EWS-FLI1 sites using STAG2 or RAD21—an essential subunit of the cohesin tripartite ring—ChIP-seq binding profiles generated from A673 cells. Here, we uncovered that STAG2-cohesin loading was markedly higher in the immediate vicinity of short (1xGGAA) EWS-FLI1 sites that lost binding compared to the long GGAA-repeat enhancers that gained binding following STAG2 inactivation (Fig. 2*B*). Consistently, CTCF binding was more frequently detected at monomeric GGAA elements (Fig. 2*C*), suggesting that STAG2-cohesin more frequently coloads at short GGAA-repeat enhancers where it is essential for stable binding of EWS-FLI1.

Given the established pioneer function of EWS-FLI1 in decompacting chromatin (10, 11), we next examined whether its redistribution upon STAG2 loss affected chromatin accessibility. ATAC-seq analysis revealed a widespread remodeling of open chromatin upon STAG2 inhibition, which showed a strong positive correlation (R = 0.3, *P*-value < 2.2e−16) with changes in EWS-FLI1 chromatin occupancy (Fig. 2*D*). Sites with reduced EWS-FLI1 binding also showed a decrease in DNA accessibility in the STAG2-deficient cells, whereas chromatin regions gaining EWS-FLI1 binding showed a corresponding increase in accessibility relative to the control cells (Fig. 2*E* and *SI Appendix,* Fig. S2*E*). In agreement with these accessibility patterns, H3K27ac ChIP-seq showed a significant enrichment of active histone marks at long (≥10xGGAA) microsatellite enhancers that gained EWS-FLI1 binding, accompanied by a complete loss of H3K27ac at short GGAA-repeat elements that lost binding upon STAG2 inactivation in A673 cells (Fig. 2*F*). These findings indicate that STAG2 loss selectively enhances the transcriptional potential of long GGAA-repeat microsatellite enhancers bound by EWS-FLI1. Consistent with this enhancer remodeling, many of the canonical EWS-FLI1 target genes regulated by long microsatellite enhancers (7, 30–32), including *NR0B1*, *NKX2-2*, *MEIS1, and *FCGRT**, were significantly upregulated in STAG2-deficient A673 cells relative to the control cells (Fig. 2*G*). The *FCGRT* locus exemplified these changes, containing two multimeric GGAA-repeat blocks (13x and 9x) that showed increased EWS-FLI1 occupancy and active enhancer-associated H3K27ac marks upon loss of STAG2 (Figs. 1*H* and 2*H*). Opposite epigenomic changes were seen upon STAG2 inactivation at the *TMEM233* gene locus that harbors an EWS-FLI1 enhancer site with short GGAA-repeat motifs (*SI Appendix,* Fig. S2*F*).

### STAG2 Loss Amplifies EWS-FLI1 Transcriptional Activity At Long GGAA-repeat Enhancers and Attenuates Monomeric Function.

**Fig. 3.**
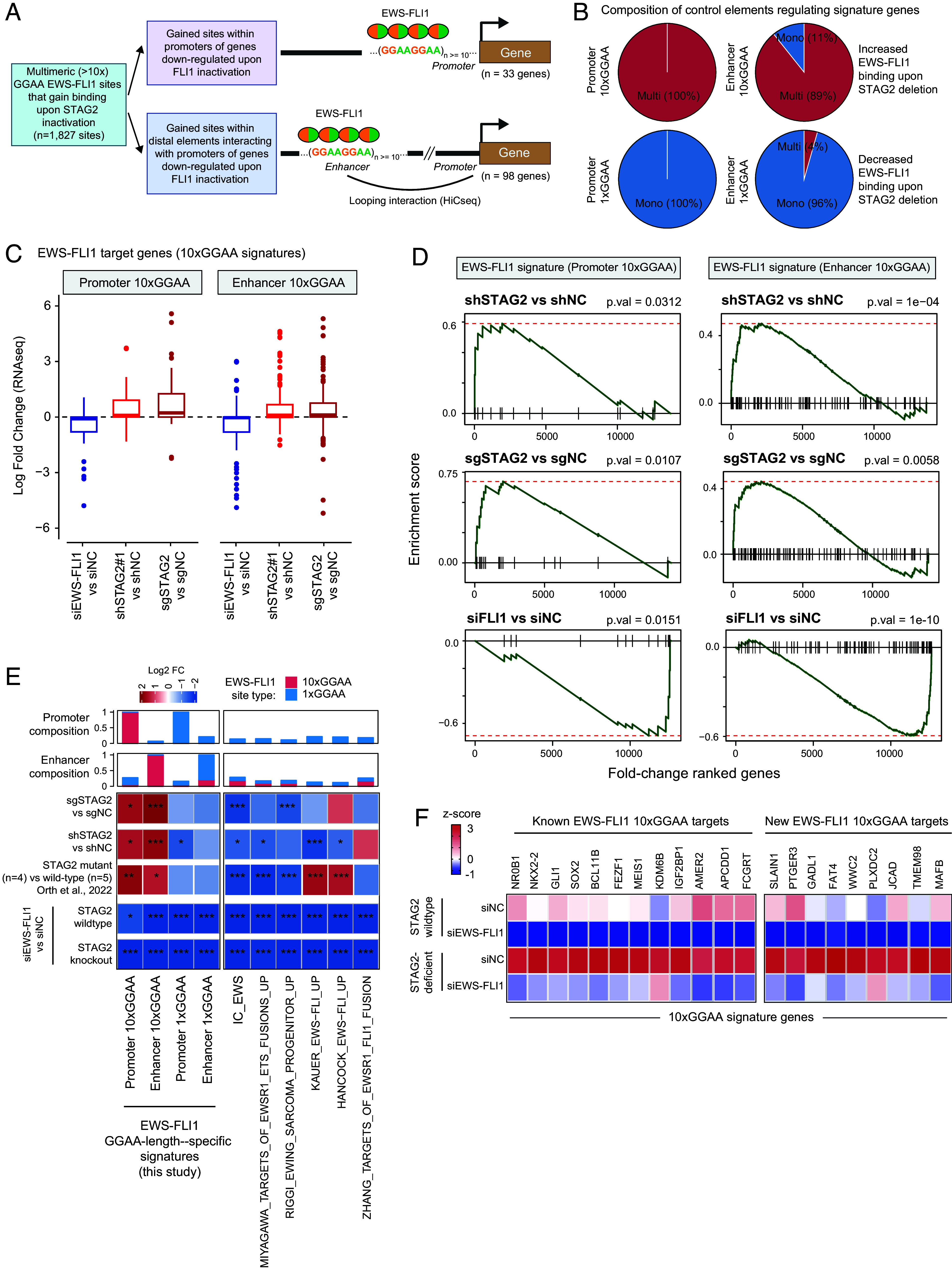
STAG2 loss amplifies EWS-FLI1 transcriptional activity at long GGAA-repeat enhancers and dampens monomeric elements. (*A*) Schematic representation of the workflow used for EWS-FLI1 10x GGAA signature definition. (*B*) Pie charts stratifying all EWS-FLI1-bound sites that make Hi-C contact with the signature genes into monomeric or multimeric GGAA enhancers. (*C*) Boxplots showing expression of the 10x promoter and enhancer signature across STAG2 knockdown, STAG2 knockout, and EWS-FLI1 knockdown conditions compared with their respective controls. (*D*) GSEA plots of promoter and enhancer EWS-FLI1 10xGGAA signatures in transcriptomes from STAG2 knockdown, STAG2 knockout, and EWS-FLI1 knockdown conditions compared with their respective controls (n = 2 biological replicates, GSEA enrichment test). (*E*) Heatmap of log2 fold-change of the 1xGGAA or 10xGGAA enhancer/promoter and published EWS-FLI1 signatures across distinct genetic perturbation groups. *P < 0.05; ***P < 0.001. (*F*) Heatmap of z-score normalized expression (RNAseq) of known and putative 10x GGAA target genes in wildtype or STAG2-deficient A673 cells treated with control or EWS-FLI1-targeting siRNA (n = 2 replicates).

To delineate transcriptional consequences of the altered EWS-FLI1 GGAA binding preference in STAG2-deficient cells, we integrated remodeled enhancer sites with Hi-C-derived enhancer–promoter interaction maps to identify their direct gene targets (Fig. 3*A* and *SI Appendix,* Fig. S3*A*). We further refined these candidates by intersecting them with genes that are transcriptionally activated by the EWS-FLI1 protein using RNA-seq data generated from STAG2-null or wildtype A673 cells following siRNA-mediated inhibition of EWS-FLI1 function (*SI Appendix,* Fig. S3*B*). This strategy enabled us to define four distinct gene signatures corresponding to EWS-FLI1 activation via either 1xGGAA or 10xGGAA binding events at promoter or enhancer control elements (Dataset S1).

To validate these signatures, we mapped all EWS-FLI1-bound *cis*-regulatory elements making direct contact with the promoter region of constituent genes using published Hi-C data (33) and, based on the GGAA-repeat lengths, assigned them to either monomeric or multimeric (≥5xGGAA) enhancer classes. This analysis revealed 10xGGAA signature genes to be predominantly regulated by long multimeric GGAA-repeat enhancers, whereas the majority of distal interactions to 1xGGAA target genes originated from monomeric EWS-FLI1 binding sites (Fig. 3*B* and *SI Appendix,* Fig. S3*C*). Next, using RNA-seq data (7), we confirmed that both the short and long GGAA-repeat signatures were robustly reactivated upon exogenous reintroduction of EWS-FLI1 in A673 cells in which endogenous EWS-FLI1 expression had been silenced (*SI Appendix,* Fig. S3*D*).

Having credentialled repeat-length–specific EWS-FLI1 up-regulated transcriptional signatures, we next evaluated their regulation across STAG2-deficient and wild-type contexts. As anticipated, EWS-FLI1 inhibition led to a marked depletion of both the 10xGGAA promoter and enhancer signatures (Fig. 3*C*). In contrast, however, shRNA-mediated STAG2 inhibition or CRISPR-mediated STAG2 deletion triggered a strong activation of EWS-FLI1 targets regulated by the long microsatellite enhancers (Fig. 3 *C* and *D*). Using microarray data from the Ewing Sarcoma Cell Line Atlas (29), we also found that STAG2 mutant cell lines (n = 4) had a positive enrichment of both the 10xGGAA enhancer and promoter signatures relative to STAG2 wildtype lines (n = 5) (*SI Appendix,* Fig. S3*E*). Notably, restoration of STAG2 activity either through viral STAG2 overexpression in the CRISPR-knockout or doxycycline washout in the inducible shSTAG2 A673 models led to a significant downregulation of EWS-FLI1 10xGGAA gene targets (*SI Appendix,* Fig. S3 *F*–*J*). This STAG2-dependent EWS-FLI1 transcriptional reprogramming was also observed in STAG2-mutant TC-32 cells, where STAG2 reexpression led to a significant downregulation of a subset of 10xGGAA target genes (*SI Appendix,* Fig. S4*A*). In contrast to long-microsatellite EWS-FLI1 targets, 1xGGAA-regulated genes were uniformly down-regulated upon loss of either EWS-FLI1 or STAG2 function in A673 cells (*SI Appendix,* Fig. S4*B*), indicating selective attenuation of monomeric enhancer activity. Notably, consistent with previous reports (27, 28), most published EWS-FLI1-upregulated gene signatures showed a modest, and in some cases significant, down-regulation in STAG2-deficient cells relative to controls (Fig. 3*E* and *SI Appendix,* Fig. S4 *C* and *D*). Together, these findings demonstrate that STAG2 loss does not globally diminish EWS-FLI1 activity but rather redistributes and amplifies its function at long GGAA-repeat microsatellite enhancer, with a parallel attenuation of transcriptional activity at monomeric sites.

While the EWS-FLI1 10xGGAA signature consisted of many established microsatellite-regulated targets, it also uncovered several previously unrecognized genes that are under dominant transcriptional regulation of long GGAA-repeat enhancers (Fig. 3*F* and *SI Appendix,* Fig. S5*A*). Consistently, these genes were up-regulated upon STAG2 inactivation but were significantly down-regulated upon EWS-FLI1 inhibition regardless of the STAG2 functional status in A673 cells (Fig. 3*F*), further confirming their EWS-FLI1-dependent up-regulation in STAG2-deficient Ewing sarcoma cells. While STAG2 loss led to the downregulation of neuronal differentiation and synaptic function pathways, the EWS-FLI1 10xGGAA genes showed significant enrichment for developmental pathways, including corticospinal tract morphogenesis and neural crest and mesenchymal cell development (*SI Appendix,* Fig. S5 *B* and *C*). Collectively, these findings suggest that STAG2 loss reprograms Ewing sarcoma cells toward a more primitive and migratory neuronal state, partly driven by EWS-FLI1 redistribution to longer GGAA-repeat enhancers.

### EWS-FLI1 10xGGAA Signature Is Elevated in Clinically Aggressive and STAG2-Altered Ewing Sarcomas.

Loss-of-function alterations in the *STAG2* gene or reduced STAG2 expression have been associated with adverse clinical outcomes in Ewing sarcoma (26, 34). To explore whether the EWS-FLI1 10xGGAA transcriptional signature amplified upon STAG2 loss could serve as a prognostic biomarker, we integrated our signature with patient survival data. Specifically, we intersected the 10xGGAA signature genes with transcripts showing a positive Cox regression coefficient for overall survival in a clinical Ewing sarcoma cohort (35), yielding a refined list of 55 EWS-FLI1 10xGGAA-regulated genes associated with poor clinical outcome (*SI Appendix,* Fig. S6*A* and Dataset S2).

**Fig. 4.**
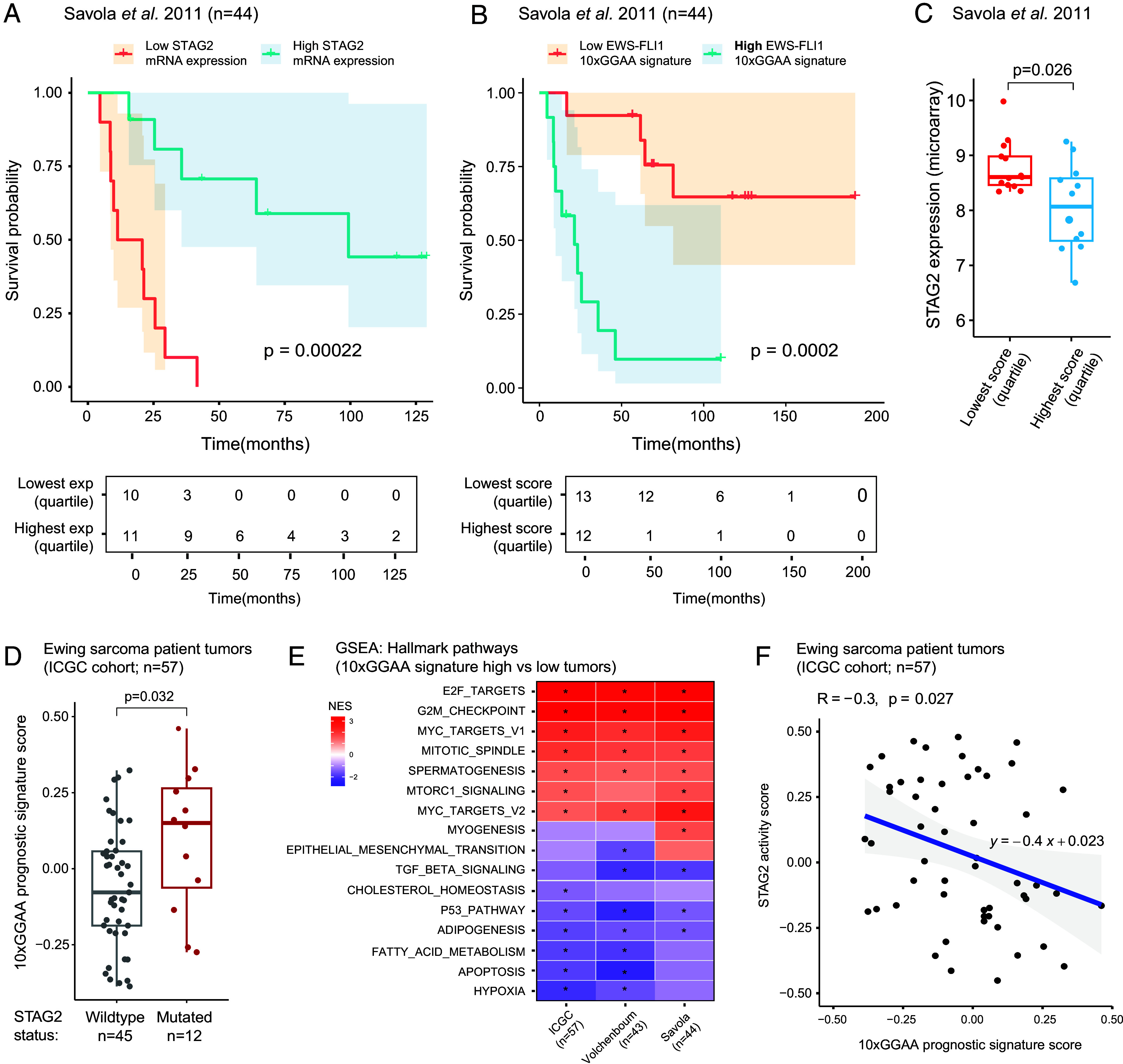
EWS-FLI1 10xGGAA gene signature is enriched in STAG2-deficient and clinically aggressive Ewing sarcomas. (*A*) Overall survival probability of patients based on quartile-based stratification of the *STAG2* mRNA levels. Patient data from Savola et al. (35, n = 44 samples). (*B*) Overall survival probability of patients based on quartile-based stratification of the 10xGGAA EWS-FLI1 oncogenic signature. Patient data from Savola et al. (35, n = 44 samples). (*C*) Boxplots showing *STAG2* mRNA expression in the 10xGGAA signature low and high groups from panel *B*. (*D*) Boxplots of the 10xGGAA EWS-FLI1 prognostic signature in STAG2 wildtype and mutant tumors. Patient data from the ICGC cohort (n = 57 tumors). (*E*) Heatmap of net enrichment scores of the hallmark pathways in 10xGGAA signature high vs low patient tumors in various clinical cohorts. *P < 0.05. (*F*) Correlation plot of the STAG2 activity and 10xGGAA prognostic signature scores in patient tumors from the ICGC cohort (n = 57, Pearson correlation test).

Consistent with prior observations, low *STAG2* expression was associated with significantly shorter overall survival (Fig. 4*A*). In contrast, elevated expression of the EWS-FLI1 10xGGAA prognostic signature identified a high-risk patient subgroup, with a median survival of fewer than 25 mo (Fig. 4*B*; *P* = 0.0002). Notably, expression of this EWS-FLI1 signature inversely correlated with *STAG2* transcript abundance (R = – 0.42, *P* = 0.0016; *SI Appendix,* Fig. S6*B*), with 10xGGAA signature-high tumors exhibiting significantly lower *STAG2* transcript levels compared to signature-low cases (Fig. 4*C*). A similar trend was observed in an independent validation cohort (36) (*SI Appendix,* Fig. S6 *C* and *D*), and in a combined dataset comprising 87 patients, where cases with high scores for the EWS-FLI1 10xGGAA signature showed significantly worse prognosis relative to the low-scoring tumors (*P* = 0.0002; *SI Appendix,* Fig. S6*E*). This suggests that the EWS-FLI1 10xGGAA signature could serve as a biomarker for risk stratification of Ewing sarcomas, where higher signature expression is associated with a more clinically aggressive disease.

To further substantiate these findings in patient samples, we analyzed RNA-seq data from 57 primary Ewing sarcoma specimens (4, 37). Through RNA-based mutation calling (see Methods), we first identified cases harboring deleterious alterations in the *STAG2* gene. Using single-sample gene set enrichment analysis, we then calculated enrichment scores for the 10xGGAA signature for each sample. STAG2-altered cases exhibited a significantly higher expression of the EWS-FLI1 10xGGAA prognostic signature compared with wild-type tumors (Fig. 4*D*). Furthermore, comparison of global transcriptomes between high-scoring versus low-scoring patient tumors revealed a significant enrichment for hallmark pathways associated with aggressive tumors, such as G2M checkpoint, E2F targets, mitotic spindle, and MYC targets, with a parallel suppression of adipogenesis and apoptosis cell-death signaling pathways (Fig. 4*E*). Similar results were seen when segregating patient tumors into high and low-scoring groups for the 10xGGAA prognostic signature using two independent microarray expression datasets (35, 36).

Next, to quantify STAG2 functional activity, we derived a STAG2-inactivation gene signature from the differential expression analysis of STAG2-silenced A673 and TC-71 cells relative to controls. As expected, this signature confirmed a significant attenuation of STAG2 activity in STAG2-mutant cell lines, compared to wild-type controls (*SI Appendix,* Fig. S6*F*). In patient tumor transcriptomes, STAG2 activity displayed a significant inverse correlation with the EWS-FLI1 10xGGAA prognostic signature (R = – 0.3, *P* = 0.027; Fig. 4*F*). Genes comprising this signature were consistently upregulated upon STAG2 inactivation and in STAG2-mutant cell lines, while being downregulated following EWS-FLI1 inhibition in A673 cells (*SI Appendix,* Fig. S6*G*). Importantly, genetic depletion of either STAG1 or CTCF did not induce the EWS-FLI1 prognostic gene program (*SI Appendix,* Fig. S6*H*). Together, these findings demonstrate that loss of STAG2-cohesin is uniquely required for hyperactivation of EWS-FLI1-driven long GGAA-microsatellite enhancers, and the associated gene signature defines clinically aggressive Ewing sarcomas.

## Discussion

The cohesin complex has long been recognized for its fundamental roles in sister chromatid cohesion, maintenance of genome integrity, organization of three-dimensional chromatin architecture, and enhancer-mediated gene regulation (14–18). However, the contribution of its subunit STAG2 to cancer pathogenesis—particularly in Ewing sarcoma—has remained widely debated and incompletely understood. Here, by systematically investigating the interplay between STAG2 and the EWS-FLI1 fusion oncoprotein, we uncover an unexpected mechanistic insight: loss of STAG2 drives a length-dependent redistribution of EWS-FLI1 toward long GGAA-repeat microsatellite enhancers, resulting in selective amplification of its oncogenic transcriptional program associated with adverse clinical outcomes.

Our results reveal that STAG2-cohesin is an essential determinant of EWS-FLI1 enhancer selection across the genome. Loss of STAG2 decreases EWS-FLI1 occupancy at short (1–4x GGAA) elements, while markedly enhancing its binding at long microsatellite enhancers harboring multiple blocks of ≥10xGGAA repeats, including those regulating canonical EWS-FLI1 microsatellite targets such as *NR0B1*, *NKX2-2*, *MEIS1*, and *FCGRT*. Cohesin-binding data placed STAG2 preferentially at monomeric GGAA elements in Ewing sarcoma cells, often in conjunction with RAD21 and CTCF, suggesting that STAG2-containing cohesin facilitates EWS-FLI1 occupancy at short GGAA enhancers, and regulates their transcriptional activity likely by stabilizing enhancer–promoter loops, as reported in earlier studies (26–28). It is important to note that while the loss of EWS-FLI1 binding was majorly restricted to short GGAA repeats, the residual cistrome in STAG2-deficient cells retains many short microsatellite enhancers, with some even showing modest gains in EWS-FLI1 binding. Further investigation is required to understand how these retained 1-4xGGAA elements differ from those that lose fusion binding, and whether STAG1-cohesin compensation drives this divergence. Additionally, it remains to be determined how STAG2-mediated reprogramming affects genes normally repressed by EWS-FLI1 that have previously been linked to binding at shorter GGAA repeats (11). Upon STAG2 loss, the EWS-FLI1 DNA-binding profile shifts markedly toward longer microsatellite-type enhancers, where cohesin complexes are infrequently cobound. This redistribution suggests that EWS-FLI1 long microsatellite sites likely operate independently of cohesin’s anchoring function to regulate target gene expression.

Importantly, EWS-FLI1 cistromic reprogramming is specific to the loss of STAG2-cohesin, as neither STAG1 loss nor CTCF depletion induces the associated transcriptional rewiring. This underlines the nonredundant and disease-specific role of STAG2 in shaping EWS-FLI1 enhancer usage and transcriptional program, which likely explains the recurrence of genetic alterations exclusively in this cohesin subunit in patient tumors. There are no mutations in the paralogous *STAG1* gene, and STAG1 expression does not have prognostic significance in Ewing sarcoma patients (3, 4, 12). Additionally, a recent publication showed STAG2 loss to reconfigure composition of the cohesin complex and reduce the overall chromatin-bound cohesin in Ewing sarcoma cells (26). This raises a critical mechanistic question: despite STAG1 and STAG2 sharing ~70-75% amino acid identity and conserved functional domains, why does STAG1-cohesin fail to compensate for STAG2 in mediating EWS-FLI1-associated functions, and how does this relate to GGAA-repeat length? Future structural and biochemical studies—potentially probing paralog-specific interactions with EWS-FLI1 or GGAA-microsatellite sequence—could illuminate these disease-specific subunit distinctions.

Contrary to earlier reports suggesting that STAG2 inactivation broadly attenuates EWS-FLI1-driven transcription (26–28), our study reveals that loss of STAG2 reprograms the EWS-FLI1 regulatory network toward long microsatellite neoenhancers that exhibit increased transcriptional activity. This apparent discrepancy can be easily explained; most published EWS-FLI1 target gene signatures likely capture its transcriptional output from short (1–4) GGAA-repeat enhancers, which constitute 90 to 95% of the EWS-FLI1 neocistrome. Consistently, our 1xGGAA signatures display a loss of expression upon STAG2 inactivation across model systems. In contrast, the EWS-FLI1 10xGGAA gene signature defined in this study provides direct evidence for a gain-of-function effect at long microsatellite enhancers following STAG2 loss. This signature encompasses both canonical and previously unrecognized EWS-FLI1 long GGAA-repeat target genes that are selectively activated upon STAG2 depletion, suppressed upon restoration of STAG2, and strictly dependent on EWS-FLI1 activity. Considering the reversibility of this reprogramming alongside the heterogeneous pattern of STAG2 protein expression observed in clinical Ewing sarcoma specimens (3, 26, 38), it is tempting to speculate that dynamic epigenetic modulation of STAG2 expression or activity could drive intratumoral cell state plasticity, thereby generating distinct subpopulations of cells with differing metastatic and survival capacities.

**Fig. 5.**
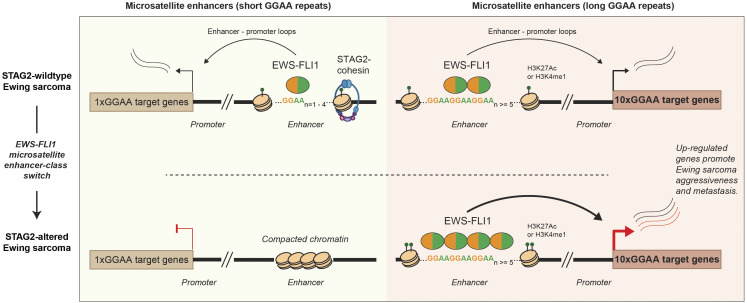
Model depicting distinct effects of STAG2 loss on EWS-FLI1 binding at short and long GGAA-repeat enhancers in Ewing sarcoma. STAG2 loss reprograms the EWS-FLI1 cistrome by altering its binding preference to GGAA-repeat elements, a phenomenon described as an “enhancer-class switch.” In STAG2-wildtype Ewing sarcoma, disruption of STAG2 activity redirects EWS-FLI1 chromatin occupancy toward long (≥5×GGAA) microsatellite enhancers, which exhibit increased chromatin accessibility and elevated H3K27ac enrichment. The heightened activity of these long GGAA-repeat enhancers reinforces a high-risk EWS-FLI1 transcriptional program associated with metastatic and aggressive disease.

Notably, unlike previously reported STAG2-loss-associated prognostic signatures that are depleted in aggressive tumors (26, 28), the EWS-FLI1 10xGGAA prognostic signature defined in this study is enriched in Ewing sarcomas with adverse clinical outcomes. Integrating these insights, we propose a unified model in which STAG2 loss triggers an EWS-FLI1 enhancer-class switch that selectively amplifies high-output long microsatellite enhancers promoting aggressive Ewing sarcoma phenotypes (Fig. 5). By uncovering this epigenomic remodeling of EWS-FLI1 neoenhancers, this study provides a mechanistic basis for the increased aggressiveness of STAG2-deficient Ewing sarcomas. Overall, this study establishes STAG2-cohesin dysfunction as an epigenetic driver of oncogenic transcriptional reprogramming, distinct from its generic association with chromosomal instability.

## Materials and Methods

### Cell Lines.

The cell lines used in this study were obtained from the American Type Culture Collection (ATCC) and cultured according to ATCC protocols. A673 cells were grown in DMEM medium with 10% fetal bovine serum (FBS; Invitrogen). TC-71 cells were grown in IMDM media supplemented with 10% FBS. A humidified incubator with 5% CO_2_ was used to grow cells. Cell line genotyping was performed every fortnight, while *Mycoplasma* testing was performed once a month. The Profiler Plus machine at the University of Michigan Sequencing Core was used for this. The resulting data were compared to short tandem repeat profiles available in ATCC to authenticate their identity.

### Antibodies.

For immunoblotting, the following antibodies were used: STAG2(CST:5882S); EWS-Fli1 (Abcam:ab15289);H3 (Cell Signaling Technologies: 3638S); GAPDH (Cell Signaling Technologies: 3683); STAG1 (Abcam:ab4457); H3K4me1 (Abcam:ab8895); H3K27ac (Abcam:ab4729).

### Immunoblotting.

Cell lysates were prepared as described previously (39). Briefly, RIPA buffer (Thermo Fisher Scientific, 89900) with Halt™ Protease and Phosphatase Inhibitor Cocktail (Thermo Fisher Scientific, 78440) was used to lyse the cells, followed by denaturation at 70 °C for 10 min, followed by measuring protein concentration using the Pierce 660 nm Protein Assay (Thermo Fisher Scientific, 22660). Between 10 to 30 μg of total protein per sample was resolved on either NuPAGE 3 to 8% Tris-Acetate or NuPAGE 4 to 12% Bis-Tris gels (Thermo Fisher Scientific), transferred to nitrocellulose membranes (Thermo Fisher Scientific, 88018) using a semidry transfer system (Trans-Blot Turbo; Bio-Rad). Membranes were blocked for 1 h in TBS containing 0.1% Tween-20 (TBS-T) and 5% nonfat dry milk, then incubated overnight at 4 °C with primary antibodies. For the secondary antibodies, we used HRP-conjugated antibodies from Bio-Rad at a 1:20,000 dilution in TBS-T for 1 h at room temperature. This was followed by signal detection and visualization on an Odyssey CLx Imager.

### siRNA/shRNA-Mediated Gene Knockdown.

Cell lysates were prepared as described previously (40). Briefly, cells were seeded at a density of 300,000 cells per well in a 6-well plate, followed by transfection with 25 nM of SMARTpool ON-TARGET plus siRNAs or nontargeting controls using the RNAiMAX reagent 12 h later. Total protein was extracted 72 h later to confirm efficient knockdown of the target genes. Catalogue numbers and guide sequences (5’ to 3’) of siRNA SMARTpools (Dharmacon) used are nontargeting control (cat. no. D-001810-10-05); STAG2:(Cat#L-021351-00-0005); STAG1:(Cat#L-010638-01-0010); FLI1:(Cat#L-003892-00-0005). For shRNA-mediated loss of STAG2, we used doxycycline-inducible shRNAs from Dharmacon shRNA#1: TCGAATTTCAGCTATCGCA and shRNA#2:ATCTCGGTCATGCTTGTAT. STAG2 shRNAs were packaged into lentiviruses to generate doxycycline-inducible stable lines. For all downstream functional sequencing assays, A673 cells were treated with doxycycline for 10 d while TC-71 cells were treated for 5 d.

### CRISPR-Cas9-Mediated Gene Knockout.

Cells were seeded at a density of 300,000 cells per well in a 6-well plate. The following day, cells were transfected with lentiCRISPR-V2 plasmids encoding either a nontargeting (sgNC) or an sgRNA targeting STAG1 or STAG2. This was followed by 3 d of puromycin selection, after which functional downstream assays were performed. The lentiCRISPR-V2 vector was a gift from Dr. Feng Zhang’s lab (Addgene plasmid # 52961). The following sgRNA sequences were used:

sgNC#1:5′-GTAGCGAACGTGTCCGGCGT-3′;sgNC#2:5′-GACCGGAACGATCTCGCGTA-3′sgSTAG2#1:5’CACCGTTGGTGATGACCATTCATT3’;sgSTAG2#2:5’CACCGTCATCAACTTCATAGCTGCC3’;sgSTAG2#3:5’CACCGATTTCGACATACAAGCACCC3’

### ATAC-seq.

ATAC–seq libraries were generated using a modified transposition protocol based on Buenrostro et al. (41). In brief, 50,000 cells were rinsed in ice-cold PBS and gently permeabilized in cytoplasmic lysis buffer (CER-I, NE-PER kit, Invitrogen, 78833) on ice for approximately 5–8 min with intermittent mixing to release nuclei. Nuclei were collected by centrifugation at 1,300 g for 5 min at 4 °C and resuspended in 50 μL of 1× TD buffer containing Tn5 transposase (Nextera DNA Library Preparation Kit, FC-121-1031) for 30 min at 37 °C. Transposed DNA was immediately purified with a Qiagen MinElute column, and sequencing adapters were enriched by PCR using NEBNext High-Fidelity 2× PCR Master Mix (NEB, M0541L), with the number of amplification cycles empirically determined by qPCR to prevent overamplification. Libraries were further purified using a combination of Qiagen MinElute cleanup and SPRI bead-based size selection (Beckman Coulter, A63881), and the final ATAC–seq libraries were sequenced on an Illumina HiSeq 2500 instrument using 125-bp paired-end reads.

### ChIP-seq.

ChIP–seq was performed using Diagenode iDeal ChIP–seq kits optimized for transcription factors or histones, with minor adaptations from previously described protocols (42, 43). For each experiment, cross-linked chromatin from approximately 2 × 10^6 cells (transcription factor ChIP) or 1 × 10^6 cells (histone ChIP) was prepared according to the manufacturer’s guidelines. Cells were collected, rinsed in PBS, fixed in formaldehyde, quenched with 1.25 M glycine (1:10 of the culture volume), and lysed prior to sonication in a Diagenode Bioruptor to generate DNA fragments of ~200 bp. Immunoprecipitations were carried out overnight at 4 °C using 4 μg of antibody for transcription factor ChIP or 2 μg for histone ChIP, and immune complexes were washed and eluted according to the kit protocol, with DNA purification using the iPure kit V2 (Diagenode).

Sequencing libraries were prepared from 1 to 10 ng of ChIP DNA with an Illumina-compatible library preparation workflow that included end repair, addition of a 3′ A overhang, adaptor ligation, PCR amplification, and size selection on 3% NuSieve 3:1 agarose (Lonza), followed by QIAEX II gel extraction (QIAGEN). Library size distribution and concentration were assessed on an Agilent 2100 Bioanalyzer before sequencing. Final libraries were sequenced on an Illumina HiSeq 2500 platform to generate 125-bp single-end reads.

### ChIP-seq and ATAC-seq Processing.

Alignment of ChIP-seq and ATAC-seq reads to the reference human genome (hg38) was performed using standardized pipelines in TPO (https://github.com/mctp/tpo). Briefly, reads were aligned using BWA mem with default settings (44). Trimming was performed using bbduk, and duplicates were removed using Picard. Peak calling was performed using MACS2 using default settings (45). Narrow peaks were called for transcription factor ChIP-seq data using default parameters; broad peaks were called for histone acetylation using default MACS2 parameters; and ATAC-seq peaks were called using MACS2 with the additional options “--format BAMPE”.

Cohesion site overlap comparisons were performed using bedtools, first sorting bed files, widening regions +/-250 bp via bedtools slop, merging overlapping peak regions, and then intersecting with regions of interest. Counts of peak intersections were then plotted in R using ggplot2 as pie charts. A Fisher’s exact test was also calculated in R for significance.

Locations of GGAA repeats in the hg38 genome were identified by matching the GGAA string to the BSgenomc.Hsapiens.UCSC.hg38 library. Comparisons of regions of interest were then made against this reference list in R, resulting in lists of GGAA repeats per peak, whether continuous or separated, and totals for each. These datasets were then classified into bins using the tidyverse and plotted as histograms with ggplot2 (46).

To compare EWS-FLI1 and H3K27ac ChIP-seq from STAG2 mutant and wild-type cell lines, we downloaded fastq files from the Ewing Sarcoma cell line atlas (29) and processed them through the ChIP-Seq pipeline as described above. Samples were normalized to 16 M reads across all samples, and peaks were called using MACS2. A few cell lines from the STAG2 mutant group (TC32, POE, MHHHES1, and EW24) and two from the STAG2 wild-type group (SKNMC and TC71) were excluded because their aligned read percent duplication values failed our quality thresholds, or the sample was paired-end data, which was inconsistent with the rest of the dataset. In-house code was used to classify the called peak files into nGGAA repeat categories. Barplots were generated in R using ggplot2 and tidyverse.

### RNA-seq and Analysis.

RNA-seq libraries were generated from 200 to 1,000 ng of total RNA. Poly(A)+ RNA enrichment, cDNA synthesis, end repair, addition of a 3’ A overhang, and ligation of indexed Illumina adapters were carried out following the TruSeq RNA library preparation protocol (Illumina). Resulting libraries were size-selected for cDNA fragments of approximately 250 to 300 bp using a 3% NuSieve 3:1 agarose gel (Lonza), purified with QIAEX II (QIAGEN), and subsequently amplified by PCR with Phusion DNA polymerase (New England Biolabs). Library size distribution and concentration were assessed on an Agilent 2100 Bioanalyzer. Paired-end sequencing (2 × 100 bp) was performed on an Illumina HiSeq 2500 platform, targeting a depth of 15 to 20 million paired reads per sample.

RNA-seq data were processed and analyzed using standardized pipelines in TPO (https://github.com/mctp/tpo). A general overview of its function and best practices is described in prior publications (47, 48). In addition to standard processing, variant calling on RNA sequencing data was performed using TNscope with default settings (“max_fisher_pv_active 0.05,” “min_tumor_allele_frac 0.0075,” “min_init_tumor_lod 2.5,” “assemble_mode 4,” and “trim_soft_clip”), and custom filters were applied to outputs. Differential gene expression was performed using the R Bioconductor package limma (49). Briefly, RNA count data were normalized using voom (11), and a linear regression model was fitted across sample groups using limma. Enrichment heatmaps were generated using Deeptools’ computeMatrix and plotHeatmap tools (50).

#### Signature scoring and signature-high/low group stratification.

To enable cross-platform and cross-cohort comparisons, we converted each STAG2-associated transcriptional program (see Signature definition below) into a per-sample signature score using single-sample gene set enrichment analysis (ssGSEA) (51). For RNA-seq count matrices, raw count matrices were transformed using variance-stabilizing transformation (VST, as implemented in the DESeq2 package) (52), to obtain approximately log-scale, variance-stabilized expression values suitable for ssGSEA. For microarray expression matrices, ssGSEA was applied directly to log-scale expression matrices. Concordance between signatures was assessed using Pearson correlation of per-sample scores. To define “signature-high” and “signature-low” groups consistent with the expected prevalence of STAG2 alterations in Ewing sarcoma (~25%), samples within each cohort were stratified by quantiles of the prognostic score. The top 25% were designated signature-high, and the bottom 25% were designated signature-low; the remaining samples were not used for binary differential analyses. For microarray analyses, we compared the transcriptome between STAG2 mutant (SKES1, MIC, RH1, and EW22) and STAG2 wild-type (A673, RDES, EW1, EW7, and CHLA10) cell lines. These samples were selected and credentialed based on the quality of the FLI1 ChIP-seq. Normalized data were downloaded from GSE176190 (29).

#### Differential expression analysis across platforms.

Differential gene expression (DE) between signature-high and signature-low groups was performed using data-type-appropriate models. For RNA-seq datasets with raw counts (ICGC and MI-OncoSeq), DE was computed using DESeq2 after filtering low-abundance genes (sum of counts across samples ≥ 10) and size-factor normalization. For microarray datasets (Savola and Volchenboum), DE was performed using limma with empirical Bayes moderation (49). For each cohort, ranked gene lists were generated using the DE test statistic (DEseq2 Wald statistic for RNA-seq; limma moderated t-statistic for microarray). To compare transcriptional profiles of STAG2 mutant and wild-type cell lines, processed microarray data from GSE176190 (29) were downloaded and analyzed for distinct signature activities.

#### Pathway enrichment.

Fast gene set enrichment analysis (fgsea) (53) was applied to the ranked gene lists to compute normalized enrichment scores (NES) and adjusted p-values (Benjamini–Hochberg) for MSigDB Hallmark gene sets (H) (54). Fgsea multilevel estimation was used to avoid fixed permutation constraints.

### Signature Definition.

For the multimeric 10xGGAA promoter signatures, 18400 peaks were identified from the A673 FLI1 ChIP-seqs in STAG2 wild-type and STAG2 knockdown datasets. Of these, 1960 peaks were multimeric upregulated peaks (number of consecutive GGAAs ≥ 10 and Fold > 0.5). From these peaks, 33 that overlapped promoter regions (defined as 1.5 kb upstream of TSS) constituted the promoter 10x signature.

For monomeric 1x promoter signatures, we selected 2641 peaks with GGAA = 1 and Fold < −1. Of these, 44 genes that also had significant downregulation upon siFLI1 (logFC < 0, adj.p.val < 0.05) were selected to be the 1x promoter signature.

For the multimeric 10xGGAA enhancer signature, we identified 142,247 loops in the HiC data. Of these, 16,765 loops were found in both replicates. From these loops, we identified 1036 loops in which one end overlapped an upregulated multi-GGAA FLI1 peak (Fold > 0.5), and 135 genes whose promoters map to the other end. These make up the 10x enhancer signature.

Similarly, from these loops, we identified 575 loops in which one end overlaps a downregulated mono-GGAA FLI1 peak (Fold < −1), and 107 genes whose promoters map to the other end. Of these genes, 29 were downregulated by siFLI1, and they comprise the 1x enhancer signature.

From the 10xGGAA promoter signatures and 10xGGAA enhancer signatures, genes associated with poorer prognosis in Savola et al. were selected as a prognostic signature (35).

In addition to the peak/loop-based definitions of multimeric (10x) and monomeric (1x) GGAA promoter/enhancer signatures, we established a compact, clinically oriented 55-gene prognostic score gene set as an “aggressiveness proxy”.

### Analyses of Hi-C Data.

HiC data were processed through the arima pipeline using default parameters, and hicDetectLoops was applied to detect loops using the parameters “--maxLoopDistance 5000000 --windowSize 10 --peakWidth 6”. Briefly, HiC reads were aligned to the hg38 genome, loops for each sample were detected using hicDetectLoops, and only high-confidence loops captured in replicates were used to define the gene signatures.

### Publicly Available Patient Datasets.

Data from 6 publicly available patient cohorts were used in this study, including GSE116495, GSE133228, GSE17679, GSE63157, GSE193824, and EGAD00001004493. GSE133228 and GSE116495 were utilized to validate the effect of STAG2 KO in A673 and TC71 cell lines. HiC data from GSE193824 were used to identify significant HiC loops in A673 cells. RNA-seq data from the EGAD00001004493 cohort were used to evaluate the effect of the EWS-FLI1 oncogenic fusion. Survival analyses were performed using the GSE17679 and GSE63157 datasets. Signature scores were calculated for each patient, and overall survival was evaluated using KM plots.

## Supplementary Material

Appendix 01 (PDF)

Dataset S01 (XLSX)

Dataset S02 (XLSX)

Dataset S03 (XLSX)

## Data Availability

All sequencing data generated in this manuscript have been deposited in GEO (GSE314154, GSE314152, and GSE314150). All custom codes used for data analyses are freely available from the following public repositories: Zenodo: https://doi.org/10.5281/zenodo.17991601 (55). All study data are included in the article and/or supporting information.
