# Supplementary material for: STAG2 loss amplifies EWS-FLI1-driven microsatellite enhancer activity promoting Ewing sarcoma aggressiveness
Source: Proc Natl Acad Sci U S A. 2026 Apr 8;123(15):e2537425123. doi: 10.1073/pnas.2537425123 (PMC13079922; doi:10.1073/pnas.2537425123)
Supplement: Supplementary file 1 — Appendix 01 (PDF) [file pnas.2537425123.sapp.pdf]

Supporting Information for

STAG2 loss amplifies EWS-FLI1-driven microsatellite enhancer activity promoting Ewing sarcoma aggressiveness

Sanjana Eyunni[1,2,*], Shih-Chun Chu[1,2,*], Mary L Guan[2], Michaela Louw[1,2], Eleanor Young[1,2], Sandra E. Carson[2], Jianhui Gong[2,3], Marcin Cieslik[1,2,3,4*], Arul M. Chinnaiyan[1,2,4,5,6,#], Abhijit Parolia[1,2,4,6,#].

1. Department of Pathology, University of Michigan, Ann Arbor, MI, USA.
2. Michigan Center for Translational Pathology, University of Michigan, Ann Arbor, MI, USA.
3. Department of Computational Medicine and Bioinformatics, University of Michigan, Ann Arbor, MI, USA.
4. Rogel Cancer Center, University of Michigan, Ann Arbor, MI, USA.
5. Howard Hughes Medical Institute, University of Michigan, Ann Arbor, MI, USA.
6. Department of Urology, University of Michigan, Ann Arbor, MI, USA.

*. These authors contributed equally to this work.
#. Corresponding authors

Correspondence to:
Arul M. Chinnaiyan, M.D., Ph.D.
Email: arul@med.umich.edu

Abhijit Parolia, Ph.D.
Email: aparolia@med.umich.edu

Author Contributions: A.P. and A.M.C. conceptualized the study and supervised the overall project. M.C. contributed to the design of computational analyses. A.P., S.E., and M.G. designed and performed the experiments and analyzed the data. A.P., S.E., and S.C. assembled figures and wrote the manuscript. S.C. and E.Y. performed and supported computational data analyses. J.G. helped analyze patient transcriptomic data. M.L. and S.E.C. assisted with experimental execution and next-generation sequencing assays.

Competing Interest Statement: A.M.C. is a co-founder and serves on the scientific advisory boards of Lynx Dx, Medsyn Pharma, NuLynx Therapeutics, and Esanik Therapeutics. None of these entities has licensed, supported, or is otherwise related to the work presented in this study. The remaining authors declare no competing interests.

Classification: Biological Sciences - Medical Sciences

Keywords: STAG2-cohesin, GGAA repeat enhancers, repeat-length–specific gene signatures, disease prognostication, EWS-FLI1 cistromic reprogramming.

This PDF file includes:

Figures S1 to S6

Other Supporting Information for this manuscript include the following:

Data S1 (Excel file)
Data S2 (Excel file)
Data S3 (Excel file)

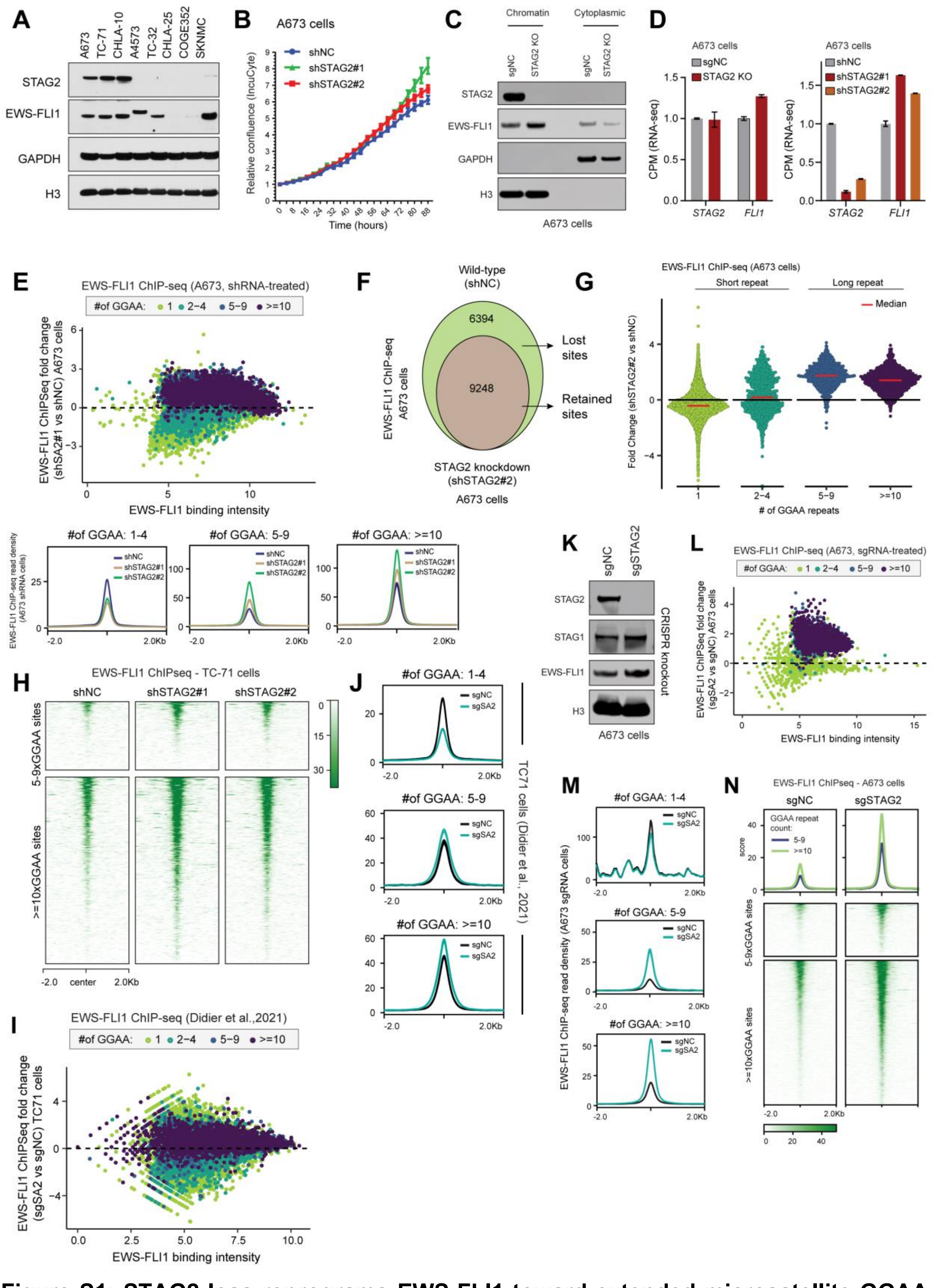

Figure S1: STAG2 loss reprograms EWS-FLI1 toward extended microsatellite GGAA-repeat enhancers. A) Immunoblot of noted proteins in a panel of STAG2 mutant and STAG2 wild-type Ewing sarcoma cell lines. **B**) Incucyte growth plots of A673 cells treated with control shRNA or two independent STAG2-targeting shRNAs. **C**) Immunoblot of noted proteins in chromatin fraction lysates of A673 control and STAG2 knockout lines. **D**) Barplots of STAG2

and FLI1 mRNA levels in A673 control and STAG2 null cells. **E**) Top: Scatter plot comparing EWS-FLI1 ChIP-seq fold change in A673 control or STAG2-shRNA cells as a function of EWS-FLI1 binding intensity, with points colored by GGAA repeat number. Bottom: Peak profile plots of EWS-FLI1 ChIP-seq centered on the GGAA microsatellite repeats. **F**) Venn diagram overlap of EWS-FLI1 ChIP-seq peaks between A673 shNC and shSTAG2#2 cells, highlighting sites retained or lost upon STAG2 knockdown. **G**) Violin plots showing fold change in EWS-FLI1 ChIP-seq signal in A673 control and shSTAG2#2-treated cell lines, stratified by GGAA repeat number. **H**) ChIP-seq read-density heatmaps of EWS-FLI1 in TC71 control cells or cells treated with two independent STAG2 shRNAs. **I**) Scatter plot comparing EWS-FLI1 ChIP-seq fold change in TC71 control or STAG2-depleted cells as a function of EWS-FLI1 binding intensity, with points colored by GGAA repeat number. **J**) Peak profile plots of EWS-FLI1 ChIP-seq centered on the GGAA microsatellite repeats from panel I. **K**) Immunoblot validating STAG2 knockout in A673 cells. **L**) Scatter plot comparing EWS-FLI1 ChIP-seq fold change in A673 control or STAG2-sgRNA cells as a function of EWS-FLI1 binding intensity, with points colored by GGAA repeat number. **M**) Peak profile plots of EWS-FLI1 ChIP-seq signal centered on GGAA microsatellites in A673 cells transduced with control or STAG2-targeting CRISPR guide RNAs. **N**) ChIP-seq read-density heatmaps of EWS-FLI1 in A673 control or STAG2 knockout cells.

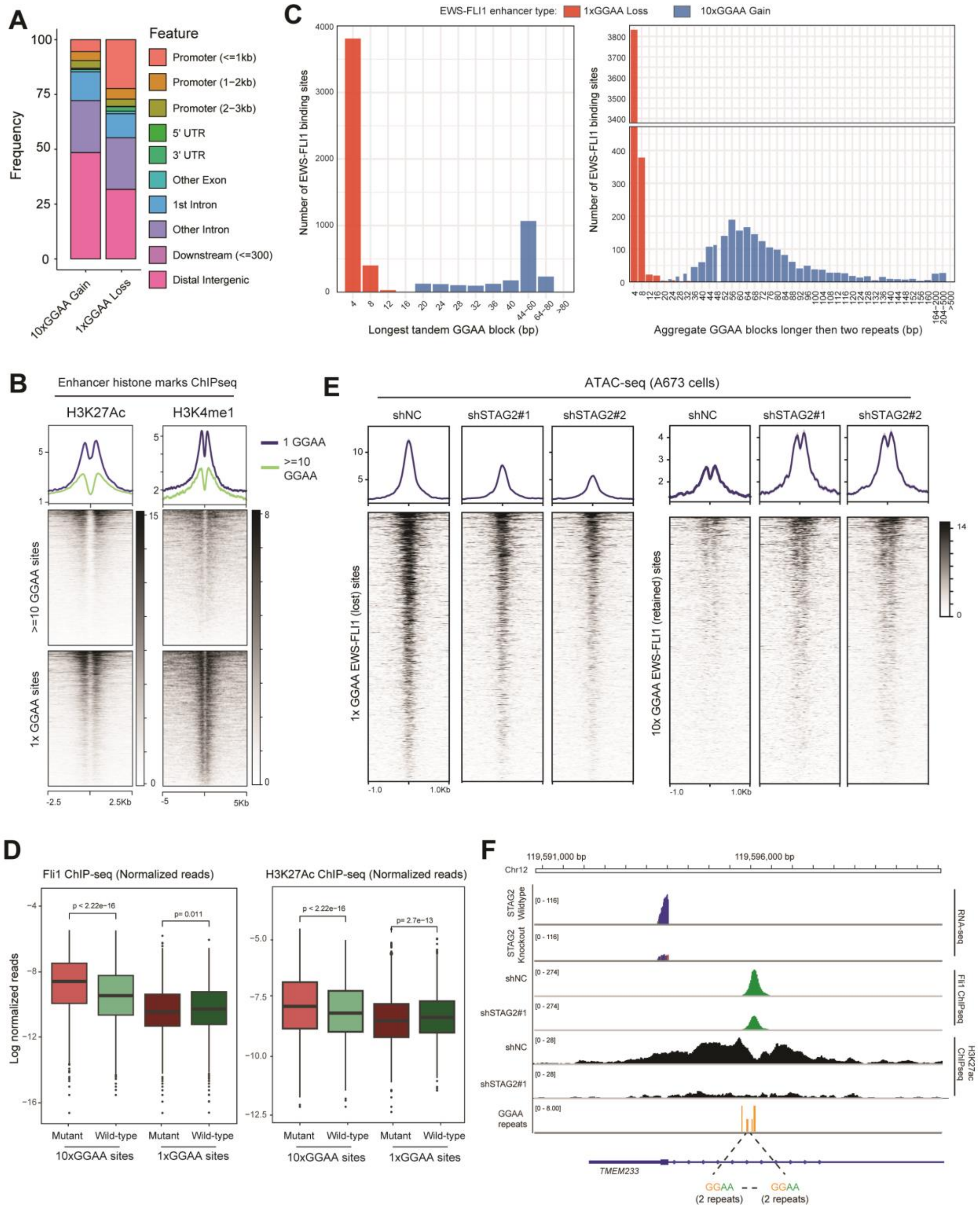

Figure S2: EWS-FLI1 reprogramming alters the epigenetic landscape at multimeric GGAA repeat elements. A) Genomic location of 10x gained and 1x lost sites defined from EWS-FLI1 ChIP-seq in A673 control and shSTAG2-treated cells. **B**) ChIP-seq read-density heatmaps of H3K27Ac and H3K4me1 at 10x and 1x EWS-FLI1-bound sites in A673 control cells. **C**) Histograms showing the distribution of EWS-FLI1-bound genomic sites stratified based on the longest GGA block (left) or aggregate GGAA blocks (right). **D**) Boxplots of normalized EWS-FLI1 and H3K27Ac ChIP-seq signal at 10x and 1x GGAA sites in STAG2 wild-type and mutant cell lines. **E**) Read-density heatmaps of chromatin accessibility (ATAC-seq) at 10x and 1x sites in A673 control cells or cells treated with two independent STAG2

shRNAs. **F**) Genome browser tracks of EWS-FLI1, H3K27ac enrichment, and RNA-seq signal at the TMEM233 (low GGAA repeat) locus in STAG2 wild-type and STAG2-depleted cells.

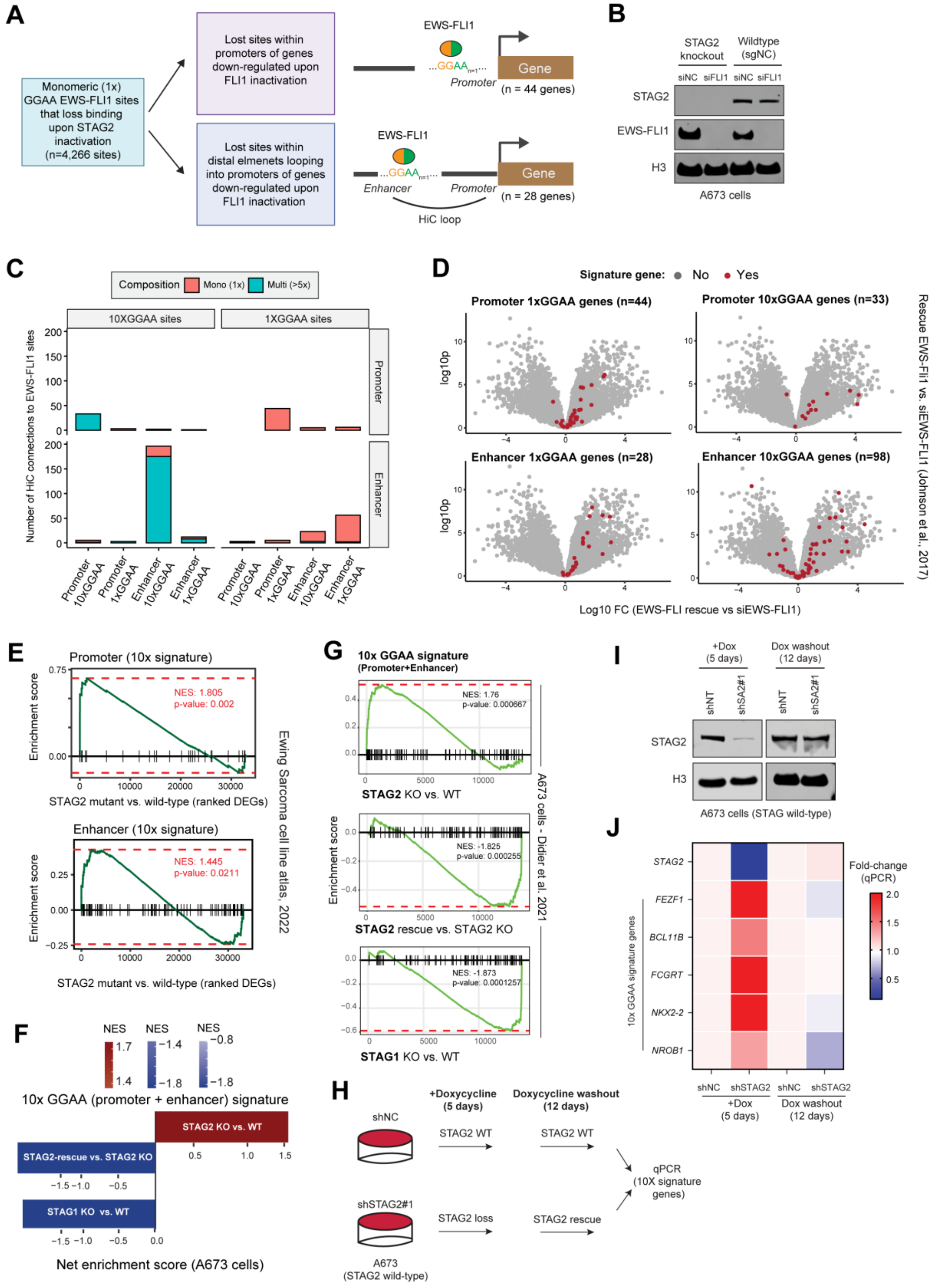

Figure S3: STAG2 loss leads to gain of EWS-FLI1 binding at multimeric 5-10xGGAA sites with downstream up-regulation of their target genes. A) Schematic representation of EWS-FLI1 1x GGAA signature definition. **B**) Immunoblot validating loss of STAG2 and EWS-FLI1 in A673 cells treated with STAG2 guide RNAs and/or EWS-FLI1-targeting siRNAs. **C**) Barplot showing distribution of monomeric or multimeric GGAA-repeats at EWS-FLI1-

bound sites that interact (Hi-C data) with promoters and enhancers of genes within different signatures. **D**) Volcano plot of up- and down-regulated genes from EWS-FLI1 rescue experiment (Johnson et al., 2017). Noted signature genes are highlighted as red dots. **E**) GSEA of the10x promoter and enhancer gene signature in microarray data comparing STAG2 mutant versus wild-type cell lines. **F**) Barplots showing net enrichment score of the 10x GGAA signature in STAG2 knockout, STAG1 knockout, or STAG2 rescue A673 cells. **G**) GSEA plots of EWS-FLI1 10×GGAA gene signatures in transcriptomes from A673 STAG2 knockout, STAG1 knockout, or STAG2 rescue conditions compared with their respective controls (n= 2 replicates, GSEA enrichment test). **H**) Schematic outline of the washout experiment. **I**) Immunoblot of STAG2 in A673 control and STAG2 shRNA lines across treatment timelines from panel H. **J**) qPCR of noted 10x-GGAA target genes in A673 control or STAG2 shRNA cells treated with doxycycline for 5 days, followed by a washout for 12 days.

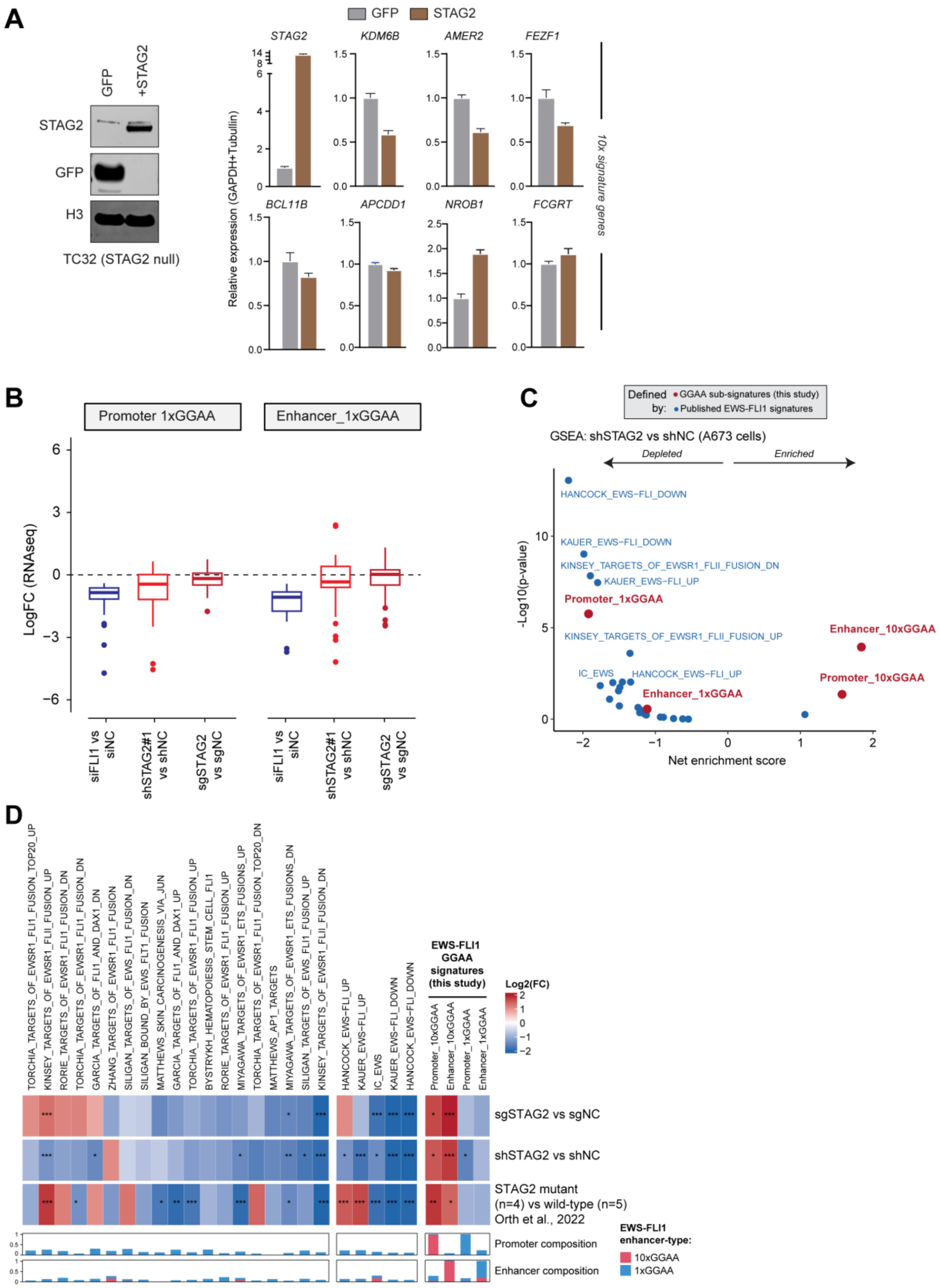

Figure S4: Gene set enrichment analysis and biological annotation of short and long GGAA-specific EWS-FLI1 gene signatures. A) Left: Immunoblot of noted proteins in TC32 cells with lentiviral overexpression of STAG2. Right: Relative expression (qPCR) of noted 10xGGAA target genes in TC32 wild-type or STAG2-overexpressing cells. **B)** Boxplots showing expression of the 1x promoter and enhancer signature across STAG2 knockdown,

STAG2 knockout, and EWS-FLI1 knockdown conditions compared with their respective controls. **C**) Volcano plot showing the net enrichment scores and statistical significance of published and in-house defined EWS-FLI1 signatures. **D**) Heatmap showing the log2 fold-change expression of distinct EWS-FLI1 gene signatures in A673 control and STAG2-deleted cells.

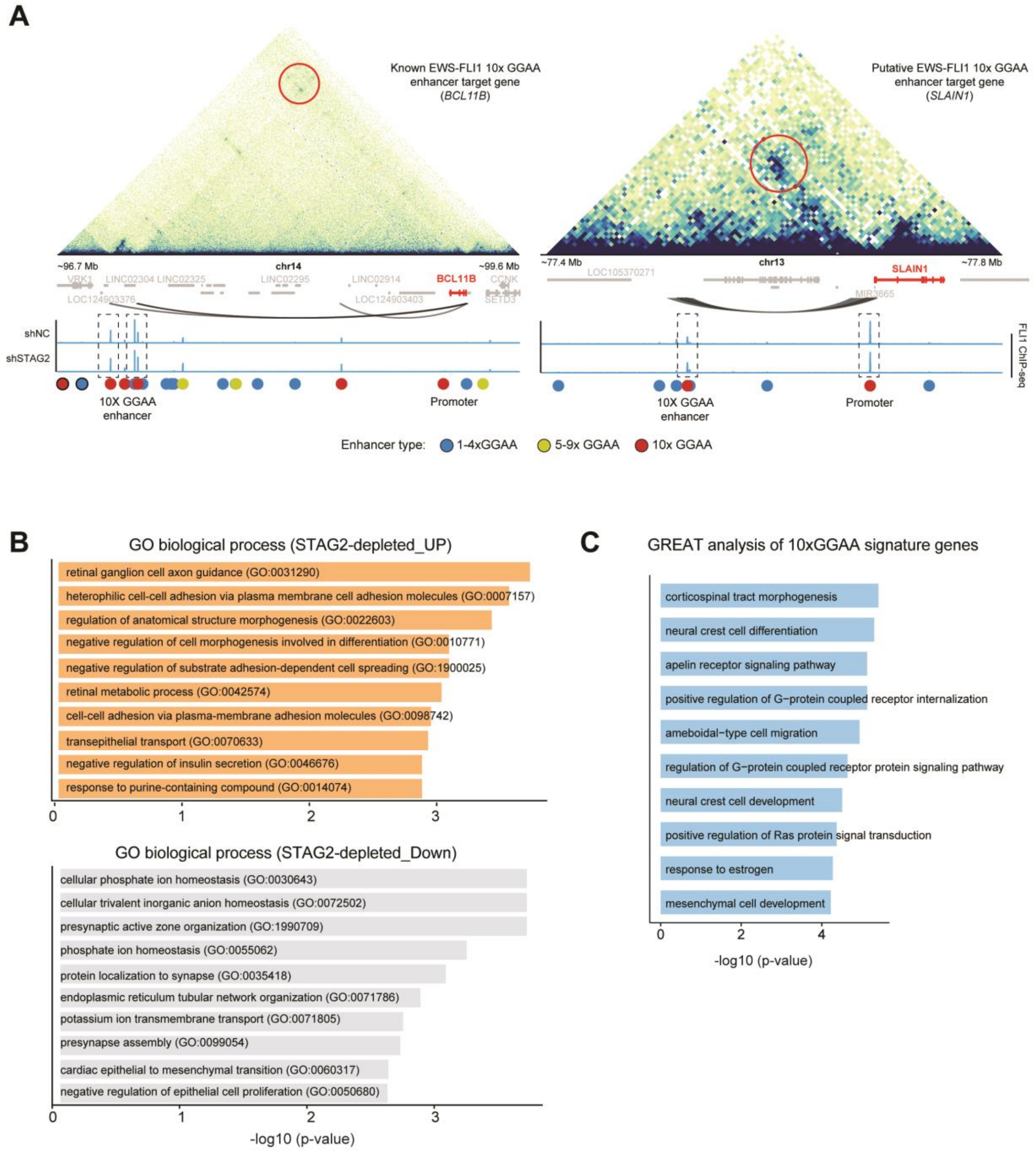

Figure S5: **A**) HiC heatmaps within the *SLAIN1* and *BCL11B* loci showing 10xGGAA enhancer-promoter looping interactions. EWS-FLI1 ChIP-seq tracks from A673 shNC and shSTAG2 cells are shown at the bottom. **B**) Enrichr analyses of genes up- and downregulated upon STAG2 loss. **C**) GREAT analyses of 10x GGAA signature genes in molecular signature and biological pathway databases.

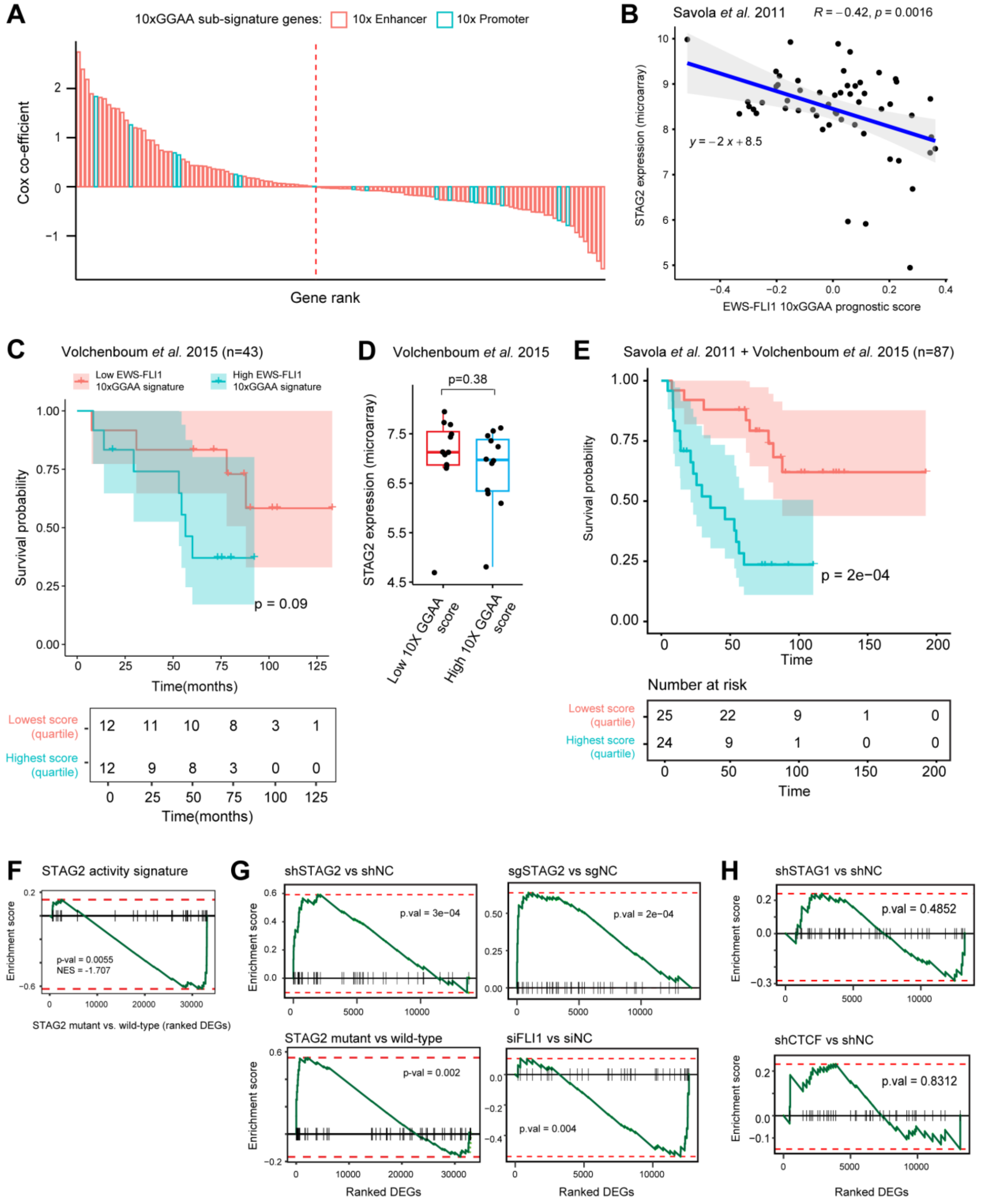

Figure S6: STAG2-loss associated EWS-FLI1 10xGGAA signature is enriched in clinically aggressive disease and STAG2-inactivated tumors. A) Cox coefficient analysis of the 10x GGAA enhancer and promoter signature. **B**) Correlation plots of the EWS-FLI1 10x prognostic signature and STAG2 expression in the Savola et al. cohort (Pearson correlation test). **C**) Overall survival probability as assessed through quartile-based stratification of the 10X GGAA signature. Patient data is collected from Volchenboum et al. (2015). **D**) Boxplots of STAG2 mRNA expression in the low and high 10XGGAA signature quartiles. **E**) Overall survival probability as assessed through quartile-based stratification of the 10X GGAA signature in the Savola and Volchenboum clinical cohorts. **F**) GSEA plots for the STAG2

activity score in microarray data from STAG2 mutant and wild-type cells (PMID: 36476851). **G**) GSEA plots for the 10xGGAA EWS-FLI1 gene signature upon STAG2 and FLI loss in A673 cells (n=2 replicates, GSEA enrichment test), or between STAG2 mutant and wildtype cell lines (PMID: 36476851). **H**) GSEA plots for the 10xGGAA EWS-FLI1 gene signature upon STAG1 or CTCF loss in A673 cells (n=2 replicates, GSEA enrichment test).

Data S1. (Excel file) Gene lists of EWS-FLI1-activated 1xGGAA and 10xGGAA enhancer and promoter signatures.

Data S2. (Excel file) Gene list of the EWS-FLI1 10xGGAA prognostic signature.

Data S3. (Excel file) Hallmark and C2 pathways that are enriched or depleted upon STAG2 loss.
